# Arylamine *N*-Acetyltransferase 2 (*NAT2*) Genetic Diversity and Traditional Subsistence: A Worldwide Population Survey

**DOI:** 10.1371/journal.pone.0018507

**Published:** 2011-04-06

**Authors:** Audrey Sabbagh, Pierre Darlu, Brigitte Crouau-Roy, Estella S. Poloni

**Affiliations:** 1 Institut de Recherche pour le Développement (IRD), UMR 216, Paris, France; 2 Université Paris Descartes, UMR 216, Paris, France; 3 UMR 7206 Eco-anthropologie et ethnobiologie, MNHN-CNRS-Université Denis Diderot, Paris, France; 4 CNRS UMR 5174 EDB, Toulouse, France; 5 Université Paul Sabatier, Toulouse, France; 6 Laboratory of Anthropology, Genetics and Peopling History, Anthropology Unit, Department of Genetics and Evolution, University of Geneva, Geneva, Switzerland; State University of New York College at Oneonta, United States of America

## Abstract

Arylamine *N*-acetyltransferase 2 (NAT2) is involved in human physiological responses to a variety of xenobiotic compounds, including common therapeutic drugs and exogenous chemicals present in the diet and the environment. Many questions remain about the evolutionary mechanisms that have led to the high prevalence of slow acetylators in the human species. Evidence from recent surveys of *NAT2* gene variation suggests that *NAT2* slow-causing variants might have become targets of positive selection as a consequence of the shift in modes of subsistence and lifestyle in human populations in the last 10,000 years. We aimed to test more extensively the hypothesis that slow acetylation prevalence in humans is related to the subsistence strategy adopted by the past populations. To this end, published frequency data on the most relevant genetic variants of *NAT2* were collected from 128 population samples (14,679 individuals) representing different subsistence modes and dietary habits, allowing a thorough analysis at both a worldwide and continent scale. A significantly higher prevalence of the slow acetylation phenotype was observed in populations practicing farming (45.4%) and herding (48.2%) as compared to populations mostly relying on hunting and gathering (22.4%) (*P* = 0.0007). This was closely mirrored by the frequency of the slow 590A variant that was found to occur at a three-fold higher frequency in food producers (25%) as compared to hunter-gatherers (8%). These findings are consistent with the hypothesis that the Neolithic transition to subsistence economies based on agricultural and pastoral resources modified the selective regime affecting the NAT2 acetylation pathway. Furthermore, the vast amount of data collected enabled us to provide a comprehensive and up-to-date description of *NAT2* worldwide genetic diversity, thus building up a useful resource of frequency data for further studies interested in epidemiological or anthropological research questions involving *NAT2*.

## Introduction

The arylamine *N*-acetyltransferase 2 (*NAT2*) gene is involved in human physiological responses to a wide range of xenobiotic compounds, including many clinically useful drugs and a variety of exogenous chemicals present in the diet and the environment. Genetic polymorphisms at the *NAT2* locus, giving rise to either the ‘slow’ or the ‘fast’ acetylator phenotype, influence individual variation in cancer susceptibility, responses to environmental toxins, and the effectiveness of prescribed medications [1], [2]. Beyond its medical relevance, *NAT2* has generated considerable interest in the field of evolutionary genetics and numerous studies have attempted to decipher the relative roles of population history and natural selection in shaping genetic variation at this locus [3]–[8].

A particularly intriguing aspect of *NAT2* gene variation is the high prevalence of slow acetylators in humans (well above 50% worldwide) which calls into question the role that slow acetylation has played in the adaptation of our species. Several non-mutually exclusive hypotheses have been proposed. A first possible explanation is that *NAT2* may be a neutrally evolving gene, the NAT2 enzyme having become dispensable or redundant with other detoxifying enzymes such as NAT1, and thus being no more essential to human life and health [7]. Under such a model, the variants conferring a slow acetylator phenotype are not more detrimental to the individual's survival than neutral polymorphisms and they may have reached high frequencies in human populations just ‘by chance’, through genetic drift. A second hypothesis invokes the action of balancing selection, favouring heterozygous individuals carrying both a fast and a slow *NAT2* allele [3], [5], [6], [8]. Many studies that used appropriate phenotyping methods have provided evidence that fast/slow heterozygotes display an acetylation activity intermediate between those of the slow/slow and fast/fast homozygotes [9]. One can thus imagine that not being a too slow or a too fast acetylator could be an advantage as compared to the two homozygotes. Finally, an alternative hypothesis involves the action of directional selection on multiple standing slow-causing variants [3], [6], [8]. The variants altering NAT2 activity may have been selectively neutral (or even slightly deleterious) and present at appreciable frequencies in human populations before becoming positively selected under new environmental conditions. Considering a global advantage of being a slow acetylator (and a roughly equivalent effect of all slow-causing mutations on phenotype and fitness), this model assumes that the different slow variants of *NAT2* may have simultaneously become targets of directional selection, thereby generating an excess of intermediate-frequency haplotypes. This complex model of ‘multiallelic’ directional selection seems to better fit the patterns of *NAT2* diversity observed in present-day populations than the standard ‘hard sweep’ model which assumes the rapid fixation of a single newly arisen advantageous mutation [10]. This, in turn, would explain why conventional tests of selection have failed to detect signatures of positive selection at the *NAT2* locus [6], [8].

The recent surveys of *NAT2* variation conducted in a large number of human populations have provided compelling evidence that at least some of the slow-causing variants of *NAT2* have been driven to present-day frequencies through the action of natural selection, although the observed patterns do not allow to discriminate between balancing selection and directional selection on multiple standing variants [3], [6], [8]. The selective advantage that a slower rate of acetylation may have conferred is thought to be a consequence of the shift in modes of subsistence and lifestyle in the last 10,000 years which triggered significant changes in dietary exposure to environmental chemicals. A diversity survey of the *NAT2* gene in six Central Asian populations has indeed revealed a clear contrast between populations having different lifestyles and dietary habits, with twice as many slow acetylators in long-term sedentary agriculturalists (55%–63%) as compared to nomadic pastoralists (26%–35%) [6]. A similar dichotomous pattern has been observed among sub-Saharan African populations, with a much higher frequency of slow acetylators in Bantu-speaking agriculturalists (46%) as compared to hunter-gatherers (10%) [4]. To further test the hypothesis that the slow acetylation phenotype may have been a key adaptation to increase our species fitness in response to the transition from foraging to farming, Luca *et al.* [8] examined *NAT2* haplotype frequencies in 47 worldwide populations (of which 12 newly studied populations), assigned them to one of the major subsistence strategies (hunter-gatherers, pastoralists or agriculturalists), and performed tests for the equality of haplotype frequencies across subsistence modes. The pool of fast haplotypes showed a strong decreasing trend in the order hunter-gatherers/pastoralists/agriculturalists, with average frequencies of 0.52, 0.36 and 0.27, respectively, significantly departing from equality (*P*<0.001). Among slow haplotypes, NAT2*5 and NAT2*6 showed a similar (inverted) trend, with significantly higher frequencies in agriculturalists (0.37 and 0.30, respectively) as compared to pastoralists (0.27 and 0.23) and hunter-gatherers (0.23 and 0.11). Luca *et al.* [8] concluded that *NAT2*-altering variants may have gained a selective advantage in populations shifting from hunting-gathering to pastoralism/agriculture and proposed the diminished folate supply resulting from the nutritional shift as a possible cause of the fitness change.

An overwhelming amount of data has been generated on *NAT2* gene polymorphisms in an impressive number of populations of distinct ethnic backgrounds since the discovery of the gene in 1990 [11]. We intended to take advantage of this large body of data to test more extensively the hypothesis that different dietary regimens and lifestyles may explain inter-population differences in *NAT2* variation. By systematically retrieving data from the literature, we collected frequency data for the most relevant genetic variants of *NAT2* in 128 population samples representing different subsistence modes and dietary habits. This allowed us to perform a thorough analysis of the covariation between *NAT2* haplotype frequencies and the main subsistence strategies at both a worldwide and continent scale. Furthermore, the vast amount of data collected provided a comprehensive and up-to-date description of worldwide *NAT2* genetic diversity, thus building up a useful resource of frequency data for further studies interested in epidemiological or anthropological research questions involving the *NAT2* gene.

## Results

**Figure 1:**
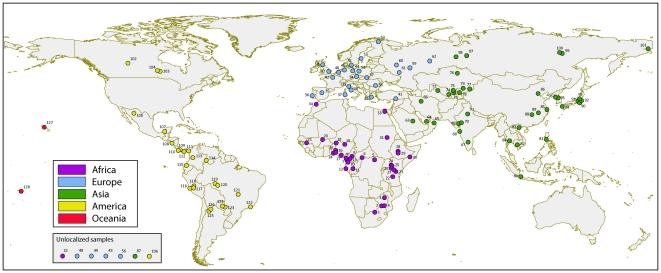
Geographic location of the 128 population samples collected from the literature. Samples are numbered as reported in Table S1 (‘sample ID’). Seven samples could not be localized on the map because of unspecified sampling location (sample 56) or because of divergence between sampling location and region of origin (samples 32, 43, 48, 49, 87, and 106); these samples are displayed in a box beneath the caption.

We created a comprehensive resource of frequency data for the seven most important genetic variants of the *NAT2* gene by systematically retrieving data from the literature (Table S1). These seven SNPs are the most commonly reported variants in surveys of *NAT2* sequence variation in human populations and their combined analysis has been shown to be highly predictive of the acetylation phenotype. In total the collected data consisted of 14,679 individuals from 128 human populations representing five continental regions: Africa (34 samples), Europe (28), Asia (39), America (25), and Oceania (2). Sample sizes ranged from 11 to 1,312 individuals, with an average of 115 (±178) individuals per sample. The number of samples genotyped for 7, 6, 5, 4 or 3 SNPs was 74, 32, 3, 8 and 11, respectively. The geographical distribution of the population samples is shown in Figure 1.

### Worldwide distribution of *NAT2* genetic and phenotypic diversity

To describe the global patterns of *NAT2* haplotype and phenotype variation, we focused on the 99 population samples adequately characterized for the seven (or six for non-African samples) SNPs of *NAT2* (see Materials and Methods). Haplotype reconstruction from the multilocus genotype data defined a total of 33 distinct *NAT2* haplotypes, whose frequencies in the entire panel are provided in Table S1, along with the number of distinct haplotypes and the within-population haplotype diversity. Ten of these 33 haplotypes are ‘private’ (i.e., only found in one population sample) and only eight occur at a worldwide frequency > 1%, among which three fast haplotypes (NAT2*4: 32.4%, NAT2*12A: 2.1%, NAT2*13A: 1.5%) and five slow haplotypes (NAT2*5B: 26.9%, NAT2*6A: 24.0%, NAT2*7B: 6.1%, NAT2*5C: 2.0%, NAT2*5A: 1.7%). African populations showed the highest level of within-population diversity (mean value of 0.79 as compared to 0.71, 0.68 and 0.70 in Europe, Asia and America, respectively) and had also the largest number of private haplotypes.

**Figure 2:**
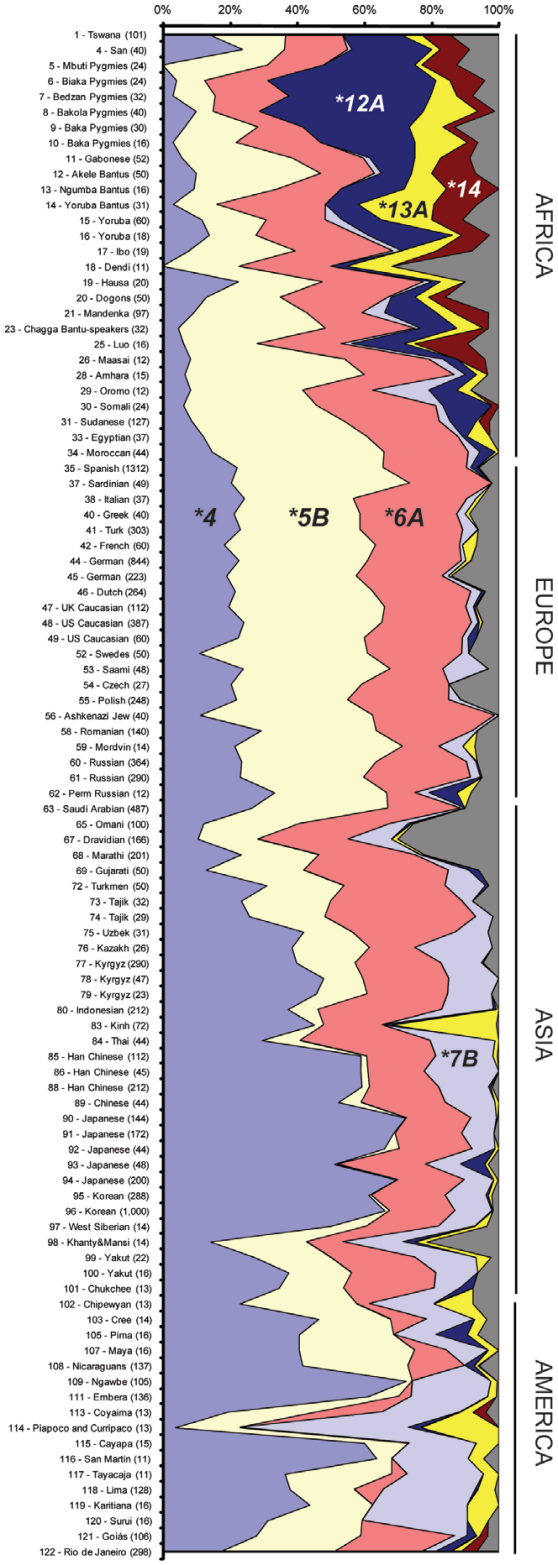
Distribution of *NAT2* haplotype frequencies in the 99 population samples included in the worldwide diversity survey. The 99 samples included 74 samples genotyped for the seven most common SNPs of *NAT2* as well as 25 non-African samples genotyped for all SNPs except the 191G>A variant. Single populations are reported on the left side of the plot, with sample ID (as reported in Table S1) preceeding the population name and sample size (number of individuals) in brackets; geographic areas are indicated on the right side. The assignment of populations to one of the four world regions was based on the origin of the population, ignoring the past 1,000 years of known human migration (e.g., people of European descent in the United States were assigned to Europe). Only haplotypes with frequencies above 5% in at least one continental region are represented individually; all other haplotypes are pooled into a single group (in grey). Also, haplotypes NAT2*14A and NAT2*14B are pooled into the NAT2*14 series.

The distribution of the most common *NAT2* haplotypes (frequency > 5% in at least one continental region) in the 99 worldwide samples revealed striking differences between continental groups (Figure 2). African populations are characterized by a low frequency of the ancestral NAT2*4 haplotype along with a high prevalence of the two other fast haplotypes, NAT2*12A and NAT2*13A, that are otherwise rare outside Africa. The NAT2*12A haplotype is particularly frequent in Pygmies and seems to be a hallmark of these populations. It is noteworthy that the haplotypes found outside Africa are essentially a subset of the collection of those found in Africa. In European populations, the derived haplotypes NAT2*5B and NAT2*6A associated with the slow acetylation phenotype are largely predominant over the fast NAT2*4 haplotype. The level of differentiation between populations was surprisingly low among Europeans (*F_ST_* = 0.003, *P* = 0.002), pointing to a remarkable homogeneity for *NAT2* variation in this continent. This sharply contrasted with the high level of population differentiation observed in Asia (*F_ST_* = 0.107, *P*<10^−5^) and America (*F_ST_* = 0.086, *P*<10^−5^), the African samples displaying an intermediate value (*F_ST_* = 0.035, *P*<10^−5^). The magnitude of frequency differences among American populations can be easily explained by the presence of both several small isolated populations undergoing rapid evolution through genetic drift (e.g. Karitiana and Surui) and a few large urban populations that probably include a substantial level of recent European and/or African ancestry (e.g. Rio de Janeiro). Regarding Asia, three distinct features can explain the extreme degree of interpopulation differentiation observed here: (1) the high level of diversity of Omani and Indian populations which display the largest number of distinct haplotypes at *NAT2* (two-fold higher than the worldwide average), (2) the unusually large frequency of the NAT2*13A haplotype (31%) in the Vietnamese Khin sample, and (3) the specific profile of North-East Asian populations (Chinese, Japanese, Korean) which form a remarkably homogeneous group in regard to *NAT2* haplotype frequencies (*F_ST_* = 0.003, *P* = 0.002). This group is notably characterized by frequencies of the fast NAT2*4 haplotype that are among the highest worldwide, particularly low frequencies of the slow NAT2*5B haplotype (otherwise frequent in all the other regions of the world) and a poor haplotype diversity (mean value of 0.55) with only three distinct haplotypes occurring at a frequency > 0.01. The slow NAT2*6A haplotype occurs at roughly similar frequencies all over the world (notwithstanding significant disparities within America), whereas the slow haplotypes NAT2*7B and NAT2*14 mainly cluster in specific continental regions (Asia/America and sub-Saharan Africa, respectively). The existence of NAT2*14 haplotypes in the samples from Goiás and Rio de Janeiro is consistent with the high level of African admixture present in the Brazilian population.

To assess population genetic structure, the 99 populations were grouped into four continental regions (Africa, Europe, Asia, and America). The vast majority of genetic variation was found to occur within populations (87.4%), a high proportion (8.3%) among continental groups, and a mere 4.3% among populations within groups. The global *F_ST_* value estimated for the 99 worldwide samples was of 0.126 (*P*<10^−5^), consistent with the average value estimated for the human genome [12].

**Figure 3:**
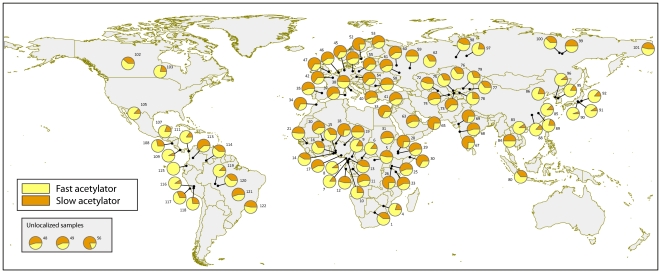
Distribution of inferred acetylation phenotypes based on genotype data. Each pie chart reports the percentage of fast (yellow) and slow (orange) acetylators in each of the 99 population samples included in the worldwide diversity survey, except for two samples where phenotype data were not available (samples 93 and 94). Intermediate acetylators were included into the fast acetylation phenotype. Three samples could not be localized on the map because of unspecified sampling location (sample 56) or because of divergence between sampling location and region of origin (samples 48 and 49); the pie charts of these samples are displayed in a box beneath the caption.

The overall population prevalence of the fast/slow acetylation phenotypes in the 99 worldwide samples investigated is reported in Table S1 and shown in Figure 3 (intermediate acetylators were pooled into the fast acetylation category). The slow acetylator status accounts for more than 50% of individuals in all populations in Europe (59% on average). While a high prevalence of this metabolic phenotype is also observed in many parts of Asia (Middle East, India, North Asia (Siberia), and Southeast Asia), this phenotype is much more rare in Northeast Asia (18% on average) owing to the high prevalence of the fast NAT2*4 haplotype in this group of populations. In Central Asia, the prevalence of slow acetylators varies greatly among populations, mainly according to lifestyle, ranging from 0.34 on average in nomad pastoralists to 0.59 on average in sedentary agriculturalists. The prevalence of slow acetylators is highly heterogeneous in Africa and in America, with striking differences among populations, even at a small geographic scale.

### Relationship between *NAT2* acetylation polymorphism and subsistence mode

Among the 128 population samples collected, 110 could be assigned to one of the three major subsistence strategies: agriculturalists (*n* = 73), pastoralists (*n* = 18) and hunter-gatherers (*n* = 19). We performed tests for the equality of haplotype frequencies across subsistence modes, considering the four main haplotype series of *NAT2* known to be associated with an altered enzyme function: NAT2*5, NAT2*6, NAT2*7 and NAT2*14, defined by the 341T>C, 590G>A, 857G>A and 191G>A slow-causing variants, respectively. The frequency of the fast acetylation phenotype, as inferred from genotype data, was also compared across subsistence categories.

Hunter-gatherers showed a significantly higher prevalence of the fast acetylation phenotype (77.6%) as compared to pastoralists (51.8%) and agriculturalists (54.9%) (*P* = 0.0007, Table 1). This higher prevalence could be mainly explained by the significantly lower frequency of the slow NAT2*6 haplotype in these populations (*P*<0.0001). By contrast, a remarkably similar pattern was observed between pastoralists and agriculturalists for both phenotype and haplotype frequencies.

**Table 1:** Test for equality of frequency of phenotype and haplotype series across subsistence modes.

	Subsistence	N	Frequency (%)^a^	*P* (Kruskal Wallis test)
Fast acetylators	Hunter-gatherer	14	77.6±12.6	0.0007
	Pastoralist	17	51.8±14.6	
	Agriculturalist	59	54.9±21.3	
NAT2*5	Hunter-gatherer	14	18.9±11.8	0.053
	Pastoralist	17	30.7±13.0	
	Agriculturalist	62	30.4±17.9	
NAT2*6	Hunter-gatherer	19	8.3±8.9	< 0.0001
	Pastoralist	18	24.9±6.4	
	Agriculturalist	73	25.4±9.6	
				
NAT2*7	Hunter-gatherer	19	14.7±14.1	0.021
	Pastoralist	18	10.8±6.3	
	Agriculturalist	73	6.5±6.7	
NAT2*14	Hunter-gatherer	13	4.5±6.6	0.341
	Pastoralist	17	2.0±5.1	
	Agriculturalist	50	2.7±4.6	

a Data expressed as mean ± standard deviation.

**Figure 4:**
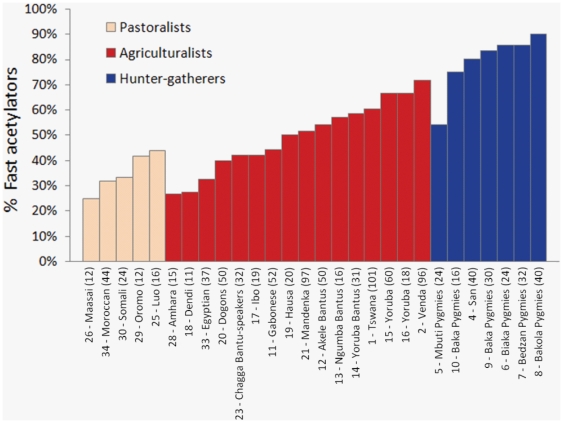
Prevalence of fast acetylators in African population samples. Samples are numbered as reported in Table S1 (‘sample ID’) and sample sizes (number of individuals) are indicated in brackets.

**Figure 5:**
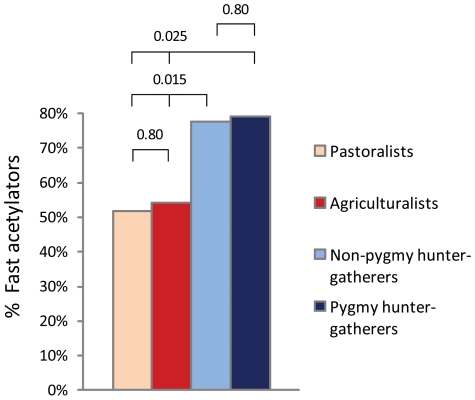
Kruskal-Wallis test for equality of frequency of the fast acetylation phenotype across subsistence modes, by distinguishing between Pygmy and non-Pygmy hunter-gatherers. Statistical significance (*P*-value) is reported above the graph.

The analysis was also performed at a smaller geographic scale, by considering only the samples from Africa (the sole continent where the three subsistence strategies co-exist in our population survey). Consistently with our previous results, a significantly higher prevalence of fast acetylators was observed among hunter-gatherers (79.1±12.0%) as compared to pastoralists (35.1±7.7%) and agriculturalists (49.1±12.8%) (*P* = 0.0005, Figure 4). However, a shortcoming of this last comparison is that the hunter-gatherers sampled in Africa are mainly represented by Pygmies which all display a high prevalence of the fast acetylation phenotype. However, at a global scale (Figure 5), we did not observe any significant difference between Pygmy and non-Pygmy hunter-gatherers (*P* = 0.80), and a higher prevalence of fast acetylators was still observed in non-Pygmy hunter-gatherers when compared to the two other subsistence groups (*P* = 0.0150).

Note that we required a minimum sample size of 10 individuals per published sample for it to be included in our database, and thus in the analyses. However, many published samples are still of very small size, thus preventing a precise estimation of allele and phenotype frequencies. Among the 110 samples included in the correlation analysis of *NAT2* polymorphism with subsistence mode, 37 include less than 30 individuals and many of these samples belong, unfortunately, to the hunter-gatherer or pastoralist categories. Consequently, even a small increase of the minimum sample size threshold implies the exclusion of many samples representing these two modes of subsistence, thereby removing the main interest of the analysis. A second round of analyses was still performed, in which only those samples including 20 individuals at least were selected. In this second round, the number of populations representing the hunter-gatherer and pastoralist modes of subsistence dropped by more than 30% (12 and 12 samples, instead of 19 and 18, respectively), but the results were similar to those obtained previously (Table S2). The significantly higher prevalence of the fast acetylation phenotype in hunter-gatherers (82.1%) than in pastoralists (54.3%) and agriculturalists (53.3%) (*P* = 0.0044, Table S2) was again observed, at the global scale, and here also this higher prevalence could be explained by the significantly lower frequency of the slow NAT2*6 haplotype in these populations (*P*<0.0001, Table S2). Similarly, within Africa, a significantly higher prevalence of fast acetylators was observed among hunter-gatherers (79.8±13.0%) as compared to pastoralists (32.6±1.1%) and agriculturalists (51.9±9.0%) (*P = *0.0036).

## Discussion

Recent genomic studies have provided growing evidence that cultural processes can have a profound impact on the human genome, triggering significant changes in allele frequencies in response to culturally modified environmental conditions [13]. Among the major human cultural transitions, the shift from an economy based on food collection (hunting and gathering) to one in which food was produced by farming and animal breeding in the Early and Middle Holocene seems to have been a major source of selection on human genes [14]. In particular, the development of agricultural subsistence systems triggered profound changes in diet and human exposure to xenobiotic compounds, bringing about a new selective regime affecting several metabolic pathways [15]. Among the most compelling examples are the genes involved in the metabolism of lactose from milk [16], [17], starch from plants [18], alcohol [19], and CYP2D6 xenobiotic substrates [20]. *NAT2* may represent a further example of a gene exposed to culturally-driven selective pressures arising from new dietary and xenobiotic exposure.

In the present study, we tested the hypothesis that the prevalence of *NAT2* slow acetylators in human populations is related to the subsistence strategy historically adopted by the past populations. Compared to the study of Luca *et al.* [8] who tested for the first time this hypothesis, our study differs in several aspects. First, the number of populations included in our analysis is much more important (110 instead of 47), with especially a 50% increase in the number of samples belonging to the hunter-gatherer and pastoralist categories (19 and 18 instead of 12 and 13, respectively). Second, in addition to *NAT2* haplotype frequencies, we analysed the prevalence of the fast acetylation phenotype as inferred from genotype data in the samples adequately genotyped for the four slow-causing variants of *NAT2*, while Luca *et al.* focused on the individual slow haplotype series and on the pool of fast haplotypes. Third, in addition to a global analysis performed at a worldwide scale, we conducted an analysis at a smaller scale, within the African continent where the three main modes of subsistence are represented in geographically close populations. Our results demonstrated a significantly higher prevalence of slow acetylators in populations practicing farming and herding as compared to populations mostly relying on hunting and gathering, thus confirming the previous findings of Luca *et al.* [8] ascertained from a smaller set of populations. However, contrary to their study, we did not observe any difference between agriculturalists and pastoralists, rather pointing to a clear contrast between food collectors and food producers. These findings are consistent with the hypothesis that the Neolithic transition to subsistence economies based on the domestication of food plants and animals modified the selective regime affecting the NAT2 acetylation pathway. On the one hand, a variety of dietary components may have lost their selective importance during the agricultural transition due to a better controlled food consumption. Thus the less crucial need to maintain a rapid NAT2-mediated acetylation activity to detoxify the poisonous xenobiotics present in wild plants might have led to a relaxation of selective pressures in food producers, leading to an increase in frequency of *NAT2* slow-acetylation alleles. However, this model supposes that *NAT2* slow variants shifted from deleterious alleles, eliminated or maintained at low frequencies through the action of purifying selection, to neutral or nearly neutral polymorphisms evolving through random genetic drift. Yet, many previous studies support an adaptive evolution of the *NAT2* gene (either due to directional or balancing selection) on *NAT2* slow-acetylation alleles, rather suggesting a selective advantage associated with a slower acetylation rate in food-producing communities [3], [5], [6], [8], [9]. Slow acetylation may thus represent a genetic adaptation to the new dietary habits and lifestyle introduced by this transition. For instance, changes in the temperature at which meat and fish are cooked modified human exposure to exogenous carcinogens, such as heterocyclic amines and polycyclic aromatic hydrocarbons, and a slower rate of acetylation might have constituted an efficient way to avoid the damaging effects of the putative carcinogens that can be activated through NAT2 acetylation. These results are in line with previous observations made in studies based on *NAT2* sequence data. Patin *et al*. [3] found evidence of a rapid increase in frequency of the NAT2*5B haplotype in Western and Central Eurasian populations in the last ∼6,500 years in response to positive selection, suggesting that this slow allele probably conferred some selective advantage to its carriers in this part of the world. While the other studies could not demonstrate clear signals of strong positive selection as those expected under the ‘hard sweep model’ using conventional approaches for detecting selection, they all nevertheless highlighted patterns of variation compatible with the action of natural selection, either in the form of balancing selection or directional selection acting on multiple standing slow-causing variants.

Gene flow restricted by geographic distance does not seem a reasonable alternative to selective pressures for explaining the observed similarities in acetylator phenotype frequencies among populations for two reasons. First, in our analysis, there is no spatial clustering of samples sharing a same subsistence mode (Figure S1): the populations are widely distributed throughout the world, making unlikely that the greater similarity in acetylation profiles between populations with a same subsistence strategy can arise from greater gene flow between them due to shorter geographic distances. For instance, hunter-gatherer populations from Africa (*n* = 7) and America (*n* = 12) are located on different continents and yet display very similar frequencies of fast acetylators (0.791±0.120 and 0.761±0.140, respectively, Mann-Whitney *P* = 0.85). Second, the contrasting pattern observed between food collectors and food producers in the prevalence of the fast acetylation phenotype was also found on a smaller spatial scale, within Africa, where the geographic distances between populations sharing the same subsistence mode are very close to those separating populations with different cultural practices. If the similarity in the prevalence of the fast acetylation phenotype between populations was mainly related to the geographic proximity of these populations due to more extensive gene flow, we would expect a positive and significant correlation between the difference in prevalence of fast acetylators and geographic distance. Yet, no such correlation was observed between the 26 African populations included in our analysis (*r* = 0.034, *P* = 0.33, Figure S2). Similarly, no correlation was neither found between the 12 populations of the American continent (*r* = −0.195, *P* = 0.91, Figure S2).

These observations do not exclude however the possibility that the greater similarity observed between populations belonging to the same subsistence category can arise from preferential gene flow between populations sharing similar cultural practices, despite geographic distance. Several examples show the influence of cultural differences on the patterns of gene flow between human populations, and thus on the patterns of genetic variation [13]. In this regard, it is interesting to note that estimates of gene flow between different Pygmy hunter-gatherer populations from Central Africa were 2.5 to 18.6 times higher than those observed between each of them and neighboring agricultural populations [21]. Of course this hypothesis does not exclude the possibility of a genetic adaptation of non-forager societies to the new xenobiotic environment introduced by the agricultural transition. It is thus possible that both selection and culturally-mediated migration may have combined to exert a strong effect on the patterns of *NAT2* genetic variation. Assessing the relative importance of these selective and non selective factors would require additional sequence variation data from the same populations at multiple independent genetic loci since different migration patterns should affect every locus in the same way, whereas selection should affect the *NAT2* locus specifically. Indeed such an approach was recently developed by Coop *et al.* [22] to assess evidence for selection at loci showing unusually strong correlations with one or more environmental variables (including subsistence variables), controlling for the effect of population structure [15].

A clear identification of the specific selective factor responsible for the change in fitness of the slow acetylation phenotype in food producers remains challenging and can hardly be addressed with the present study design. Luca *et al.* [8] proposed that the diminished dietary availability of folates consequent to the diet change in populations shifting to agriculture during the Neolithic may be a cause of the increase in frequency of *NAT2* slow haplotypes. However this hypothesis relies on the assumption that NAT2 is also involved in the metabolism of folate. Whilst several studies have convincingly demonstrated the role of NAT1 in the metabolism of the folate breakdown product p-aminobenzoylglutamate [23], [24], [25], there is still little evidence of an extra contribution of NAT2 to the overall rate of folate catabolism. Therefore, a more precise comparative analysis of populations differing by their main dietary components or xenobiotic exposure is required to determine which new or more concentrated NAT2 substrates might have been introduced in the chemical environment of food-producing communities since the transition to agriculture.

Consistently with the results of Luca *et al.* [8], the lower prevalence of slow acetylators in hunter-gatherers appeared to be mainly related to the lower frequency in these populations of the NAT2*6 haplotype series, as defined by the 590A slow-causing allele. The three-fold lower frequency of the 590A allele in hunter-gatherers (∼8%) as compared with food producers (∼25%) is in sharp contrast with the homogenous distribution reported for this variant in the 99 populations of the worldwide diversity survey (*F_ST_* = 0.02, *P*<10^−5^). Such a low level of differentiation between widely dispersed populations may be interpreted as a signature of homogenizing selection, favouring the same allelic variant in otherwise disparate populations (through either directional or balancing selection). In view of the marked correlation of the 590A variant with subsistence mode, we can speculate that the 590A slow-causing variant probably increased in frequency in populations shifting to agricultural and pastoral activities in response to new selective pressures and that the low level of differentiation observed at this locus results from the convergent selection of the 590A variant in agriculturalist and pastoralist populations which are now present in most parts of the world. Contrary to the results of Luca *et al.* [8], we did not find any significant differences in the frequencies of the NAT2*5 and NAT2*14 haplotype series across subsistence modes (*P* = 0.053 and *P* = 0.341, respectively). The significant result observed for NAT2*7 (*P* = 0.021) must be interpreted with caution due to the geographic clustering of this haplotype in Asia and America and due to its highly heterogeneous frequency among hunter-gatherers, being very rare in hunter-gatherers from Africa (0.001±0.004) and much more frequent in hunter-gatherers from America (0.19±0.11).

As a second contribution of this study, the vast amount of data collected from the literature allowed a comprehensive analysis of *NAT2* genetic diversity and provided an up-to-date picture of the global patterns of *NAT2* variation in a wide range of populations throughout the world. This considerably extended the range of our previous worldwide survey [7], bringing up to 99 the number of population samples included instead of only 28 in our previous study. In particular, the coverage of certain parts of the world, still poorly characterized until 2007, has been considerably improved thanks to many important published reports of *NAT2* genetic variation in Central, South and North Asian populations [6], [8], Native Americans [5], and sub-Saharan Africans [4], [8], [26], making *NAT2* one of the best characterized pharmacogenetic gene for interethnic and geographic variation. With this better coverage of human genetic diversity, *NAT2* variation no longer appears to be composed of discrete clusters roughly corresponding to continental regions but rather describes a broad geographic cline of allele frequencies that parallels those observed for presumably neutral genetic markers [27], [28]. In our previous survey [7], we noticed a peculiar pattern of diversity for the Thai sample as compared to other East Asian populations (Chinese, Japanese and Koreans). As this sample was the sole representative of Southeast Asian populations, we could not discriminate between a particular profile of the Thai population with respect to the *NAT2* genetic system and a true genetic differentiation between Northern and Southern East Asian populations at this locus. In the present report, the input of 25 additional populations from different parts of Asia enabled to highlight a clearly distinct pattern of diversity characterizing Northeast Asian populations (and more specifically, Chinese, Japanese and Koreans), with a particularly high prevalence of the fast ancestral NAT2*4 haplotype (accounting for more than 50% of the global variation) and a quasi-absence of the slow NAT2*5B haplotype. Besides, Thais displayed a similar profile to that seen in other populations from Southeast and Central Asia. The unexpected pattern of variation of *NAT2* in the Khin ethnic group deserves further investigations to confirm the unusually high frequency of the fast NAT2*13A haplotype in other samples from the Vietnamese population.

The specific pattern of *NAT2* haplotype frequencies in Northeast Asian populations can hardly be explained by a distinctive subsistence mode since these populations share, with those of Europe and many other parts of Asia, the same mode based primarily on agriculture. However, population-specific dietary habits and/or environmental exposures may still be valuable hypotheses to explain the specific pattern of *NAT2* haplotype frequencies in this geographic area. Unfortunately, these hypotheses cannot be tested within the framework of this study since we mainly focused on the major subsistence categories rather than on specific dietary components or xenobiotics. A dedicated study investigating *NAT2* sequence variation in Northeast Asians would be required to determine whether the increased frequency of the ancestral rapid NAT2*4 allele and/or the rarity of the slow NAT2*5B allele in these populations are the result of the action of local selective pressures or whether the observed pattern of frequencies is to be explained only by stochastic processes.

An understanding of how *NAT2* genetic diversity is structured in the human species is not only of anthropological importance, but also of medical relevance for both pharmacogenetic and epidemiological applications. For example, if major differences in allele frequencies exist between populations, individuals from different ethnic or geographic origins may respond differently to acetylated drugs. Our study confirmed a wide variation across ethnic groups in *NAT2* gene polymorphism and acetylation status at both global and microgeographic scales. The development of ethnically tailored therapies, however, appears irrelevant in the case of the *NAT2* gene polymorphism since (1) there were only few region-specific haplotypes and (2) most genetic diversity occurred between individuals rather than between populations. In this context, the ethnicity of an individual does not represent a good proxy for the acetylation status. The present report points to several geographic regions of potential interest for pharmacogenetic applications that remain poorly characterized for *NAT2* variation. A better description of *NAT2* genetic diversity in sub-Saharan African and Indian populations would be particularly interesting in view of the considerable genetic, cultural, and phenotypic variation found in these world regions. Furthermore, characterizing patterns of *NAT2* genetic diversity constitutes an imperative pre-requisite in the context of association studies aiming to better understand the role of *NAT2* in drug-induced side-effects, drug response and disease susceptibility. Spurious associations can indeed arise from an unknown population structure and significant differences in allele frequencies and haplotype structure among populations may explain some of the contradictory observations of positive, negative and no associations of *NAT2* gene polymorphisms with specific phenotypes. In this respect, the high genetic heterogeneity observed among populations from different parts of Asia, as well as among populations from Africa and America has imperatively to be taken into account when performing association studies in these populations. By contrast, the remarkable homogeneity of European populations in regard to *NAT2* allele frequencies and haplotype structure facilitates the replication of association findings across populations of European background. Interestingly, a recent report indicates that a single SNP (rs1495741), located approximately 14 kb 3′ of *NAT2*, can be substituted for the panel of seven *NAT2* SNPs, as an accurate marker of the NAT2 phenotype in molecular epidemiology studies performed in populations of European ancestry [29].

In conclusion, we provided clear evidence for a correlation between the prevalence of slow acetylators in humans and the subsistence strategy adopted by the past populations in the last 10,000 years, suggesting that a slower rate of acetylation may have gained a selective advantage in populations shifting from foraging to pastoralism/agriculture in the Neolithic period. In addition, we provided a comprehensive resource of frequency data for the most important genetic variants of *NAT2* in a large collection of human populations, allowing the investigation of specific research questions interesting both the biomedical and anthropology genetic communities.

## Materials and Methods

### Data collection

We performed an extensive survey of the literature (up to March 2010) to identify all the population samples that were genotyped for the seven most common SNPs of *NAT2* (191G>A (rs1801279), 282C>T (rs1041983), 341T>C (rs1801280), 481C>T (rs1799929), 590G>A (rs1799930), 803A>G (rs1208), and 857G>A (rs1799931)) and for which allele and/or genotype frequency data were available in the published reports. We also included population samples genotyped for only a subset of the seven SNPs of *NAT2* (at least three) only in those cases where the ethnic origin of the sample was not already represented in the set of samples genotyped for all seven SNPs. Significantly heterogenous samples (i.e., mixtures of individuals from different ethnic groups) or samples without a specific geographic or ethnic origin were excluded from the population survey, as well as samples composed of related individuals and with sample sizes below 10. In most cases, the selected samples were composed of apparently healthy, randomly selected volunteers of defined ethnicity. Information on each subject was confirmed not to be doubly included (i.e., the same individual represented in two samples). The final data set included 128 population samples from throughout the world, representing 14,679 individuals. A full description of the selected samples is provided in Table S1, along with the retrieved *NAT2* allele frequency data.

### Analysis of the worldwide distribution of *NAT2* diversity

To adequately characterize the worldwide patterns of *NAT2* gene variation, only the 74 samples genotyped for the seven common SNPs of *NAT2* were used so as to avoid possible haplotype and phenotype misclassifications due to incompleteness of genotype data. As the SNP 191G>A has been shown to be rare in non-African populations [30], we also included the 25 non-African samples genotyped for all SNPs except this one in the diversity survey, leading to a total of 99 population samples (11,286 individuals) belonging to four continental regions (Africa, Europe, Asia and America) available for analysis.

In each sample, *NAT2* haplotypes were either directly resolved using molecular-haplotyping techniques (through allele-specific PCR and restriction mapping) or computationally inferred from the unphased multilocus genotypes using statistical algorithms (based either on a parsimony, maximum-likelihood, or Bayesian approach). For some samples, a combination of the two approaches was used. The specific haplotyping method used in each sample is specified in Table S1. *NAT2* haplotypes were named in accordance with the consensus gene nomenclature of human *NAT2* alleles (http://www.louisville.edu/medschool/pharmacology/NAT2.html). The fast NAT2*4 haplotype was considered as the ancestral human haplotype, as inferred from primate sequences (unpublished data).

Thanks to the well-established genotype-phenotype correlation [31], the individual acetylation phenotype could be predicted from the pair of multilocus haplotypes carried by each subject at *NAT2*, following the acknowledged classification of NAT2* alleles into fast and slow haplotypes. The acetylation phenotype for each individual was inferred by assuming that the homozygous or compound heterozygous genotype for two haplotypes of the series NAT2*4, NAT2*11, NAT2*12 or NAT2*13 results in the rapid acetylator status, the occurrence of one of these haplotypes in combination with a haplotype of the series NAT2*5, NAT2*6, NAT2*7 or NAT2*14 results in the intermediate acetylator status and the occurrence of two haplotypes of the series NAT2*5, NAT2*6, NAT2*7 or NAT2*14 results in the slow acetylator phenotype. The proportions of slow, intermediate and fast acetylators in the 99 samples studied are provided in Table S1.

Analysis of molecular variance (AMOVA) [32], *FST* statistic [33], and measures of haplotype diversity based on estimated haplotype frequencies were computed using Arlequin v.3.11 software [34]. The molecular distance matrix (number of pairwise differences) between *NAT2* haplotypes was included in AMOVA and *FST* computations.

### Relationship between *NAT2* acetylation polymorphism and subsistence mode

The 128 collected population samples were assigned to the main subsistence mode historically practiced by the ethnic populations, using data from Murdock [35] or from the Encyclopedia of World Cultures [36] when available. Each population was classified into one of four subsistence categories: agriculturalists, pastoralists, hunter-gatherers and fishers. In 16 samples, the subsistence mode could not be reliably inferred because of a lack of information on the precise ethnic origin or ethnic composition of the sample (e.g. Iranians, Emirati, Nicaraguans, etc.). Moreover, as there were only two samples in the ‘fisher’ category (Omani and Samoans), they were discarded from analysis, leaving a total of 110 samples available for statistical analysis (see Figure S1). Homogeneity of haplotype or inferred phenotype (fast and slow acetylators) frequencies among subsistence categories was tested by the nonparametric Kruskal-Wallis test. We considered a test significant if the *P*-value was less than or equal to 0.05. The NAT2*5, NAT2*6, NAT2*7 and NAT2*14 haplotype series were represented by the 341T>C, 590G>A, 857G>A and 191G>A slow-causing variants, respectively. Data on the prevalence of the fast acetylation phenotype were considered only for the samples genotyped for all four slow-causing variants, to which we added the non-African samples genotyped for all SNPs except 191G>A (See Table S1).

## Supporting Information

Figure S1 Geographic location of the 110 population samples classified according to subsistence style. Three samples could not be localized on the map because of unspecified sampling location (sample 56) or because of divergence between sampling location and region of origin (samples 43 and 87); these samples are displayed in a box beneath the caption.
(TIF)

Figure S2 Plot of the difference in prevalence of fast acetylators between population pairs as a function of geographic distance. (A) In Africa (*n* = 26 populations). (B) In America (*n* = 12 populations). The population pairs are colour-coded (shown in the captions) according to the compared subsistence categories (A: agriculturalists; HG: hunter-gatherers; P: pastoralists). No significant correlation between geographic distance and difference in prevalence of fast acetylators was found in either one of these two continental regions (*r* = 0.034, *P* = 0.33, for Africa, and *r* = −0.195, *P* = 0.91 for America).
(DOC)

Table S1 Description of the 128 population samples collected from the literature, along with SNP, haplotype and phenotype frequencies.
(XLS)

Table S2 Test for equality of frequency of phenotype and haplotype series across subsistence modes when using only those samples with a minimum size of 20 individuals.
(XLS)
