# Supplementary figures and images for: Arylamine N-Acetyltransferase 2 (NAT2) Genetic Diversity and Traditional Subsistence: A Worldwide Population Survey
Source: PLoS One. 2011 Apr 6;6(4):e18507. doi: 10.1371/journal.pone.0018507 (PMC3071824; doi:10.1371/journal.pone.0018507)

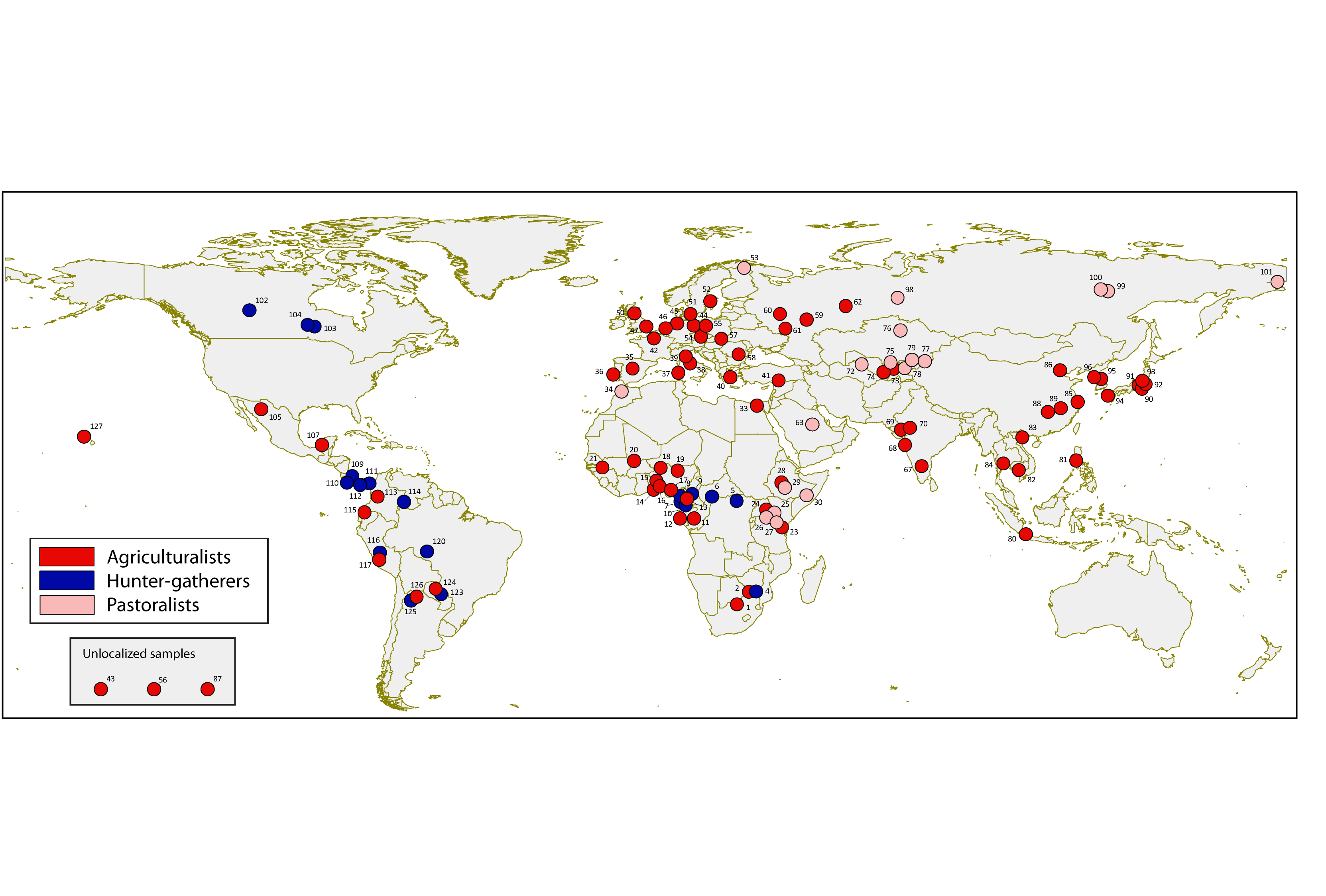

Supplement: Figure S1 — Geographic location of the 110 population samples classified according to subsistence style. Three samples could not be localized on the map because of unspecified sampling location (sample 56) or because of divergence between sampling location and region of origin (samples 43 and 87); these samples are displayed in a box beneath the caption. (TIF) [file pone.0018507.s001.tif]
